# Case Report: Adult UESL mimicking a simple hepatic cyst and initially misdiagnosed as a benign lesion

**DOI:** 10.3389/fonc.2025.1709617

**Published:** 2025-12-17

**Authors:** Yuanhang Mo, Jinrong Feng, Chunhui Liang, Xiaoqing Ma, Zongquan Wu, Xingwang Su, Shan He, Xiaoyou Xu, Yongfeng Liao, Yongdong Liu

**Affiliations:** 1 Department of General Surgery, Rongshui Miao Autonomous County People's Hospital, Liuzhou, China; 2 Department of General Surgery, Liuzhou Municipal Liutie Central Hospital, Youjiang Medical University for Nationalities, Liuzhou, China

**Keywords:** undifferentiated embryonal sarcoma of the liver, hepatic sarcoma, liver neoplasm, UESL, case report

## Abstract

We report a case of undifferentiated embryonal sarcoma of the liver (UESL) in a 50-year-old woman with no prior history of liver disease. The patient presented with abdominal distension; physical examination revealed only mild tenderness, and routine laboratory tests were within normal limits. Abdominal ultrasound and computed tomography (CT) both identified a cystic mass located in the right lobe of the liver. Initially, the patient underwent fenestration and drainage of a suspected hepatic cyst, and the histopathological analysis was consistent with a benign hepatic cyst. However, the patient was readmitted due to tumor recurrence, and a liver tumor resection was subsequently performed. The final pathological diagnosis was confirmed as UESL. Following surgery, the patient received adjuvant chemotherapy. Unfortunately, the patient passed away, with an overall survival time of 9 months. This case also provides clinicians with a profound lesson. In the face of large lesions, even when imaging suggests benign cysts, greater vigilance should be maintained regarding the possibility of malignant pathology. Every effort should be made to complete comprehensive preoperative evaluations to establish a clear diagnosis, along with meticulous perioperative planning and systematic postoperative follow-up, in order to minimize the risk of missed or delayed diagnosis.

## Introduction

Undifferentiated embryonal sarcoma of the liver (UESL) is a rare and highly malignant hepatic tumor characterized by a generally poor prognosis. It predominantly affects children between the ages of 6 and 10; however, the disease is exceedingly rare in adults, representing less than 1% of all primary liver tumors (1). First described in 1978 by Stocker and Ishak (2), UESL is now recognized as a distinct clinicopathological entity. Radiologically, this tumor typically presents as a solid mass, with or without cystic components, as observed on computed tomography and ultrasound. According to previous studies (3), UESL has a high incidence among adults around the age of 20, with 1-, 3-, and 5-year overall survival rates of 72%, 56%, and 47%, respectively. Although no clinical guidelines are currently available for the diagnosis and treatment of UESL in adults, favorable prognosis can be achieved through surgical resection combined with postoperative chemotherapy. Herein, we report a rare case of UESL, in which initial imaging and pathological findings suggested a benign hepatic cyst. The initial misdiagnosis led to the loss of the optimal treatment window, ultimately resulting in disease progression, poor prognosis, and rapid patient demise. This case underscores the importance of heightened clinical vigilance when evaluating cystic liver lesions and aims to promote accurate diagnosis to prevent similar diagnostic errors.

## Case presentation

**Figure 1:**
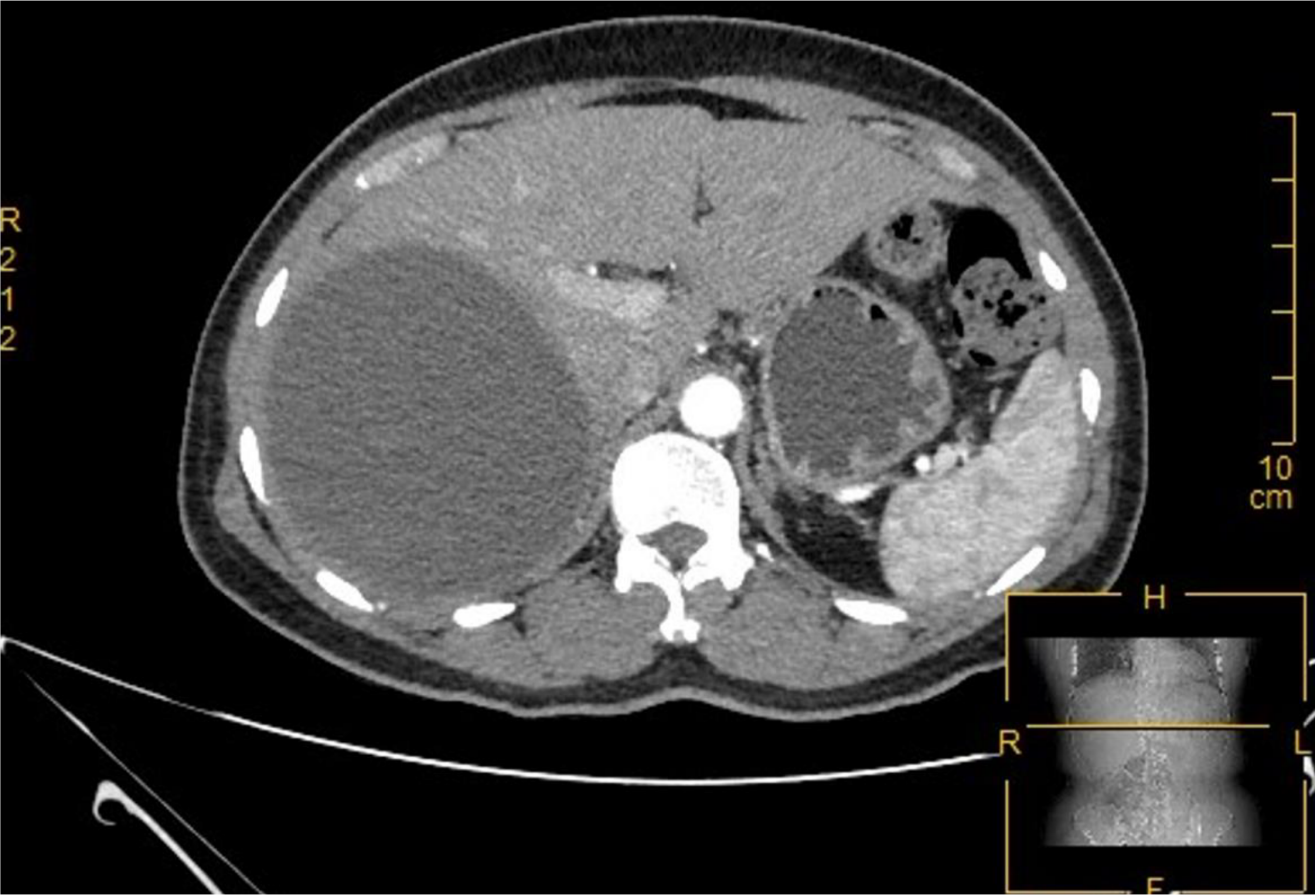
Arterial phase of enhanced CT: This was a large, round, hypodense lesion located in the right posterior lobe of the liver, measuring 10.8 cm × 9.5 cm.

The patient was a 50-year-old female with no prior history of hypertension, diabetes, hepatitis, or abdominal surgery. She had no history of smoking or alcohol abuse, no long-term medication use, and no family history of cancer or genetic disorders. Additionally, there was no occupational exposure to toxic or chemical substances, and no significant risk factors were identified. The patient presented to the Department of Hepatobiliary Surgery at our hospital with a 10-day history of abdominal distension. The physical examination revealed that the patient was alert and fully cooperative, with normal mental status. No fever or jaundice of the skin and mucous membranes was observed. Tenderness was noted in the right upper quadrant, but rebound tenderness was absent. No tenderness or rebound was noted in other abdominal regions. Mild tenderness was identified in the right lower back, with no palpable subcostal liver enlargement. Percussion over the hepatic area elicited discomfort. The patient did not experience weight loss during the three months prior to admission and presented with an ECOG performance status score of 1 upon admission. Initial laboratory analysis demonstrated normal concentrations of hemoglobin, cardiac enzymes, aspartate aminotransferase (AST), alanine aminotransferase (ALT), bilirubin, alkaline phosphatase, and tumor markers. Abdominal ultrasound examination identified a large hepatic cyst. Contrast-enhanced computed tomography (CT) of the upper abdomen revealed a large, round, low-density lesion located in the right posterior lobe of the liver. The lesion had well-defined margins, with a maximum cross-sectional size of approximately 10.8 cm × 9.5 cm. The internal density was heterogeneous, with areas of slightly increased density observed within the lesion (**Figure 1**).

**Figure 2:**
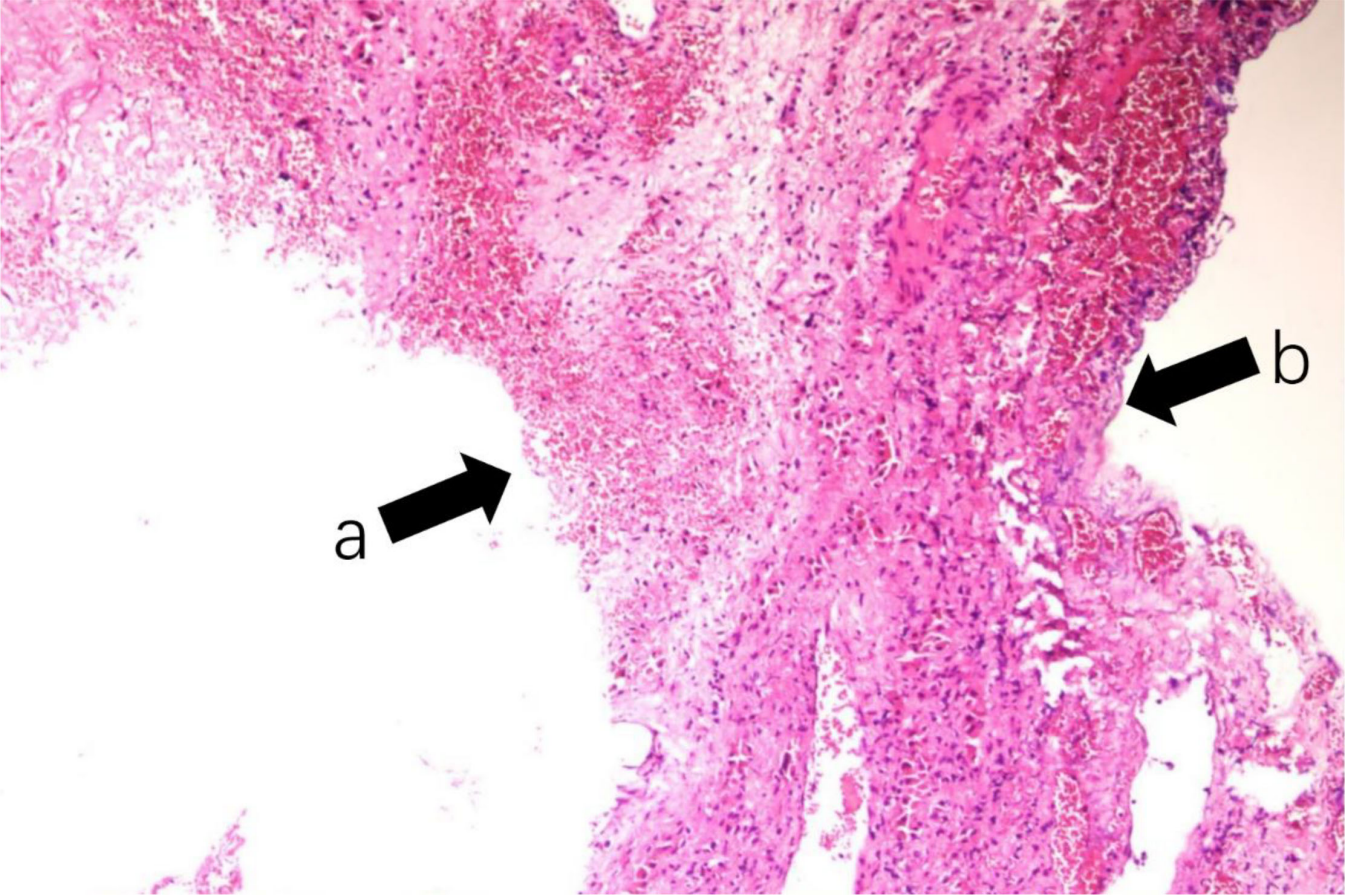
The postoperative pathological examination of the hepatic mass confirmed the presence of a hepatic cyst. The thin fibrous cyst wall (arrow a) is shown with a single layer of epithelium lining it (arrow b). There were no contents in the cyst cavity. (H&E staining, magnification: 100×).

Subsequently, the patient underwent laparoscopic fenestration and drainage of the hepatic cyst. During the operation, a cyst located in the right posterior lobe of the liver was identified as a large cyst measuring approximately 11 cm in diameter. The cyst cavity contained clear, colorless fluid, and hemorrhaging was observed in the cyst wall. Additionally, several smaller cysts approximately 2 cm in size, filled with fluid, were also noted. No abnormalities were observed in the remainder of the abdominal cavity. Approximately 1000 mL of clear cystic fluid was aspirated during the procedure. Given the absence of macroscopically evident malignant lesions, multiple tissue samples were obtained for histopathological examination, and an abdominal drainage tube was inserted at the conclusion of the operation. The postoperative pathological findings indicated hepatic cysts (**Figure 2**). The patient remained in stable condition and was discharged following the removal of the abdominal drainage tube.

**Figure 3:**
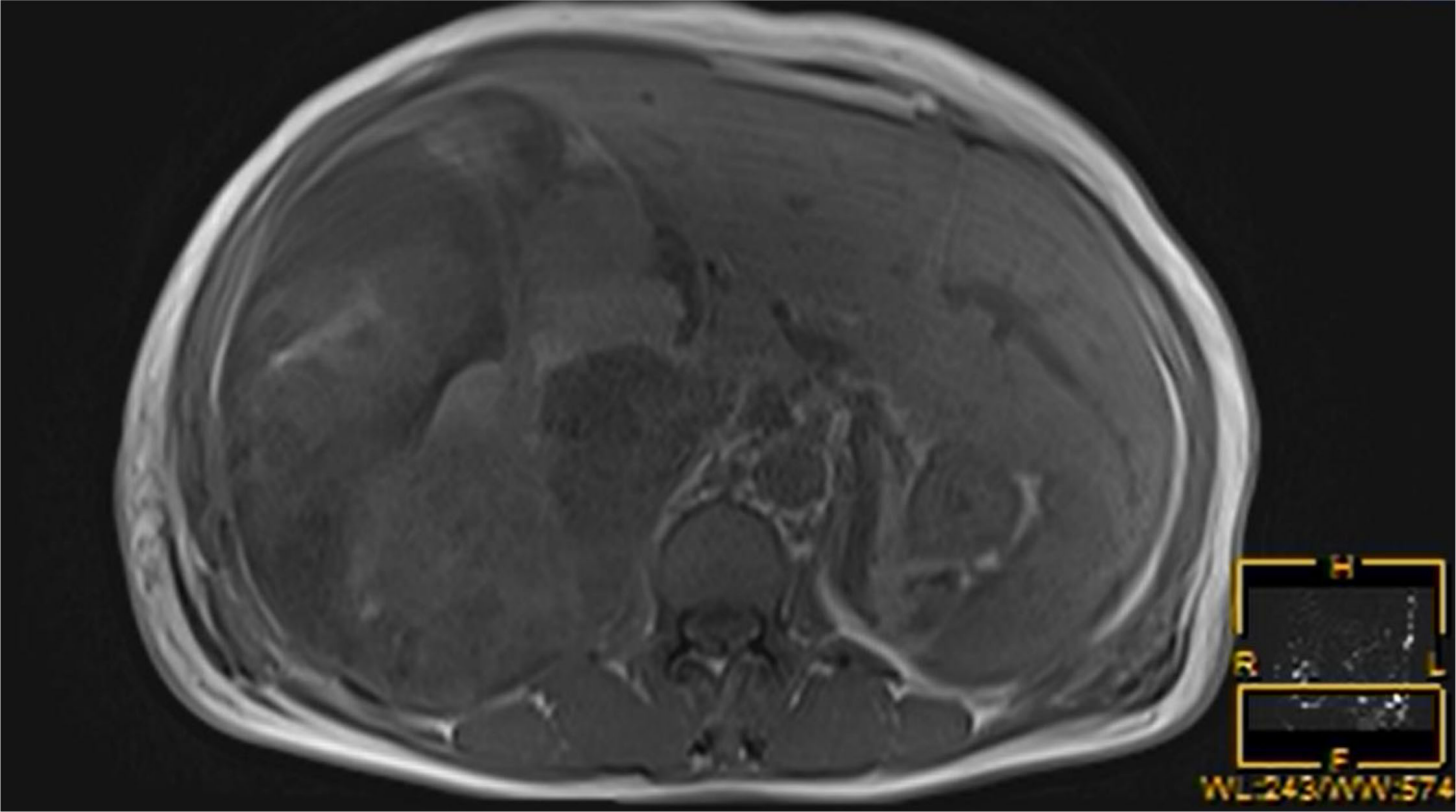
On T1-weighted magnetic resonance imaging, the tumors exhibited mixed signal intensities.

**Figure 4:**
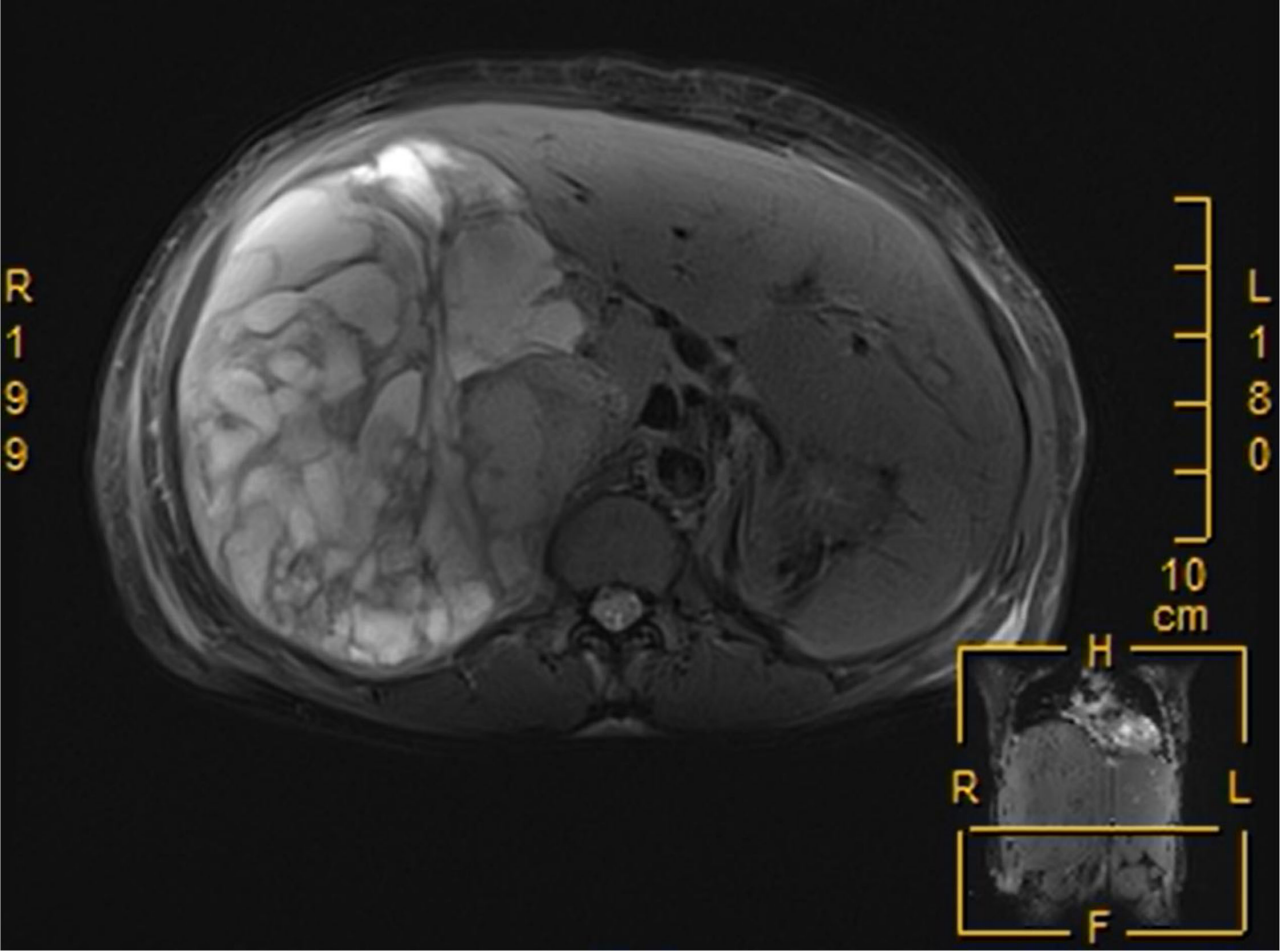
T2-weighted fat-suppressed magnetic resonance imaging predominantly revealed mixed high signal intensities.

**Figure 5:**
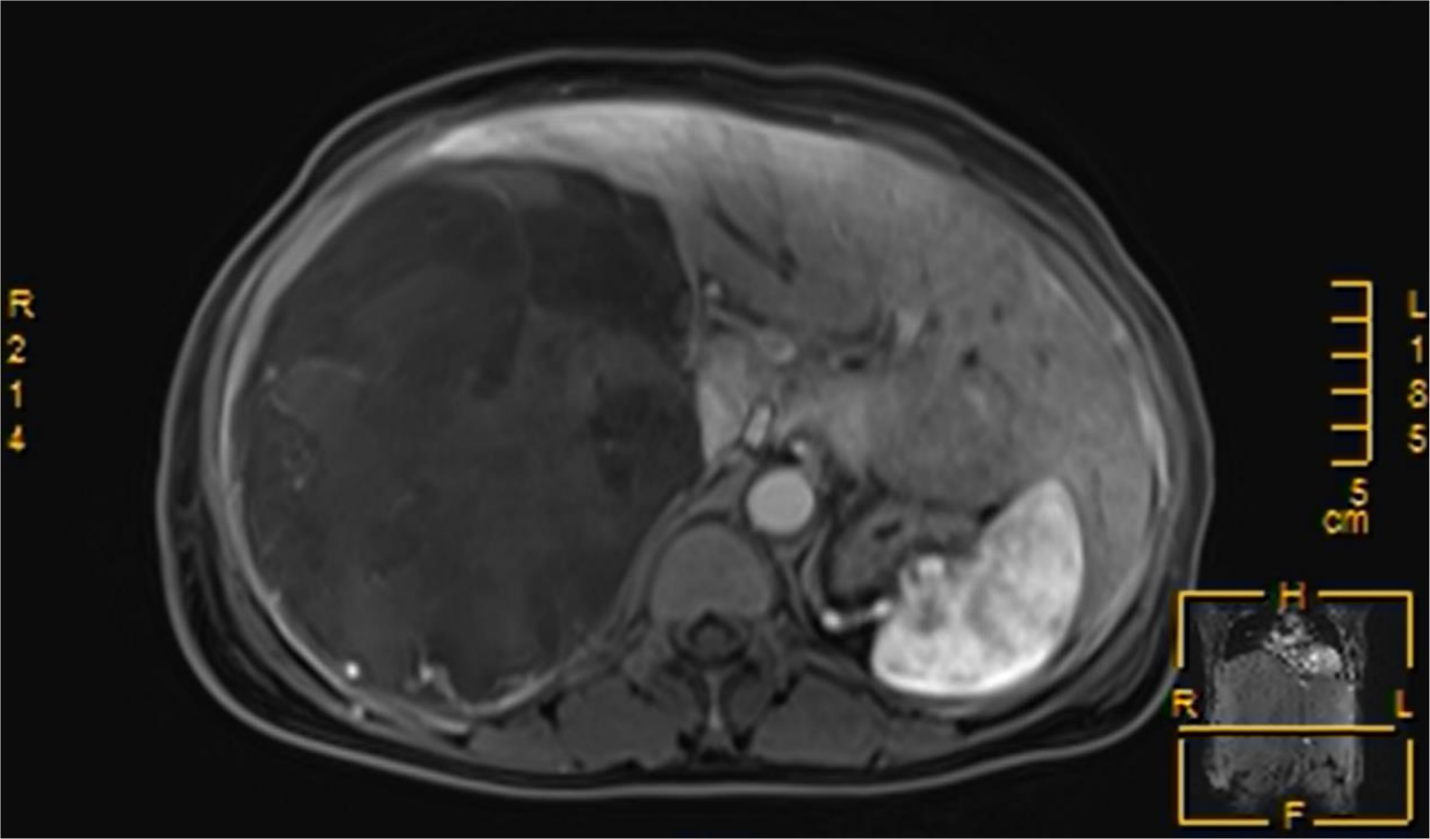
Contrast-enhanced magnetic resonance imaging demonstrated multiple patchy and linear areas of enhancement within the lesions.

Three months following discharge, the patient was readmitted to the hospital due to abdominal pain. The patient’s complete blood count revealed moderate anemia, while other laboratory tests, including ALT, AST, bilirubin levels, and tumor markers, were within normal limits, Child-Pugh liver function was classified as grade A. Abdominal magnetic resonance imaging (MRI) revealed a large, round mass with an abnormal signal located in the right hepatic lobe. The lesion exhibited heterogeneous signal intensity with multiple internal septations. The maximum cross-sectional dimension was approximately 15.8 × 12.5 cm. On T1-weighted imaging (T1WI), the mass demonstrated mixed high and low signal intensities (**Figure 3**), while T2-weighted fat-suppressed imaging showed predominantly mixed high signal intensity (**Figure 4**). Following contrast administration, multiple patchy and linear enhancing areas were observed within the lesion (**Figure 5**).

**Figure 6:**
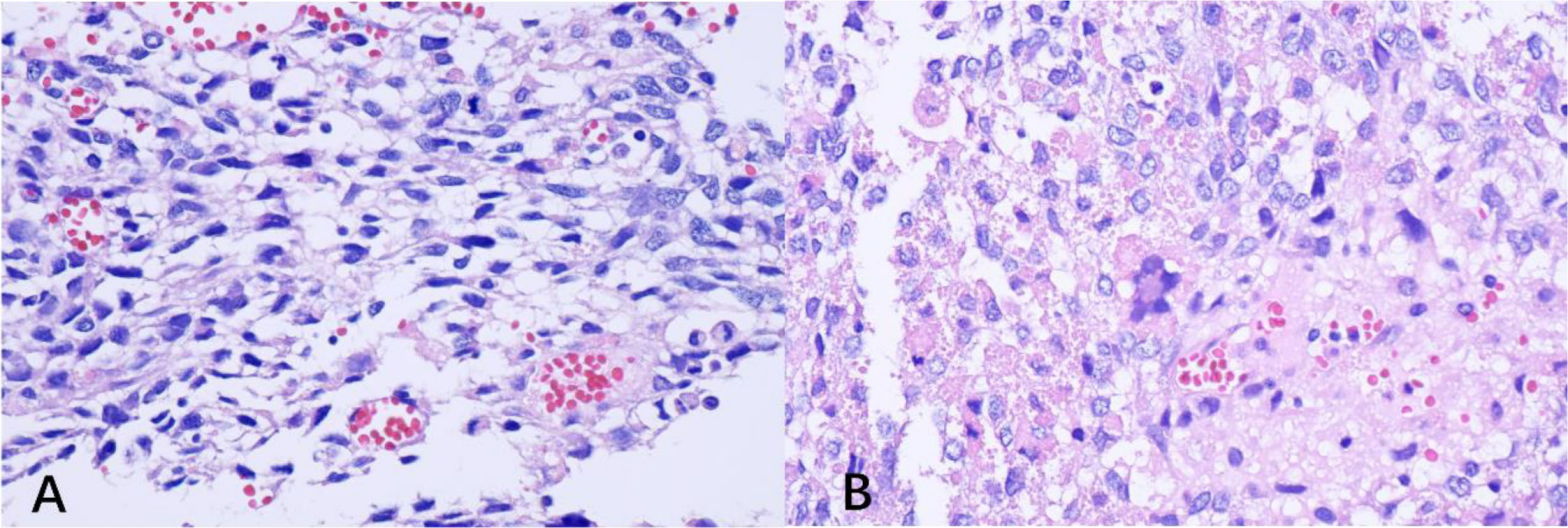
**(A, B)** represent the hematoxylin and eosin (H&E)-stained pathological sections of the postoperative tumor tissue. The lesion exhibited a loosely myxoid or densely fibromyxoid stroma, interspersed with markedly atypical neoplastic cells, frequently accompanied by areas of necrosis and hemorrhage (magnification: 200×).

**Figure 7:**
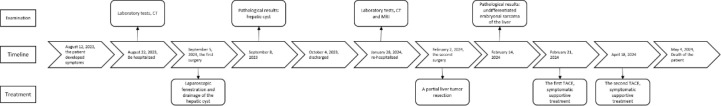
Timeline of the course of the disease in the UESL patient in this case.

The patient was assessed to have a high likelihood of hepatocellular carcinoma. After adequate preoperative evaluation, open liver tumor resection or right hepatectomy were recommended. During the operation, it was found that the tumor in the right lobe of the liver was large and adhered closely to the surrounding tissue. The tumor contained multiple cystic masses of varying sizes, with thin cyst walls appearing yellow-white in color. The intracapsular fluid was found to be partially clear and partially hemorrhagic. During the surgical procedure, a large tumor was identified with extensive adhesion to surrounding structures, including the gallbladder, colon, greater omentum, and hepatic hilum. Despite meticulous dissection, complete resection was not feasible due to tumor invasion into the hepatic hilum and the associated technical challenges in achieving safe separation. Given the duration of surgery (approximately 4 hours) and intraoperative blood loss of about 1000 mL, a decision was made to perform partial hepatic tumor resection after intraoperative assessment of patient safety and consultation with the patient’s family. Postoperative pathological examination revealed undifferentiated embryonal sarcoma of the liver (**Figure 6**), and immunohistochemistry showed Desmin(+), S-100(+), Ki67(+, 45%), CD99(+), CD68(+), CD117 (-), CD31(-), ERG (-), EMA(-), CD34(-), Vimentin(-), SMA(-), STAT6(-), MDM2(-), Actin(-), HMB45(-), MyoD1(-), Myogenin(-), CK-P(-), CD45(LCA)(-). Currently, there are no established guidelines for the diagnosis and management of UESL in adults. Based on a comprehensive review of the available literature, we consider that transcatheter arterial chemoembolization (TACE) or systemic chemotherapy following surgical resection may improve patient prognosis. After a thorough discussion of the potential benefits and risks associated with both TACE and systemic chemotherapy, the patient elected to undergo TACE. This procedure involves the use of lipiodol in combination with pirarubicin and lobaplatin, aiming to further reduce tumor burden and prolong survival. Following the laparotomy, the patient’s liver function, as assessed by the Child-Pugh classification, remained at grade B. After the initial TACE procedure, the Child-Pugh score continued to indicate class B liver function, with concomitant moderate anemia. The patient was discharged following symptomatic and supportive management. Two months later, the patient was hospitalized for the third time to undergo the second TACE procedure. MRI revealed tumor progression. Subsequently, the patient developed severe liver failure (Child-Pugh class C) accompanied by disturbances in water and electrolyte balance. Despite aggressive medical intervention, the condition did not improve, and the patient ultimately succumbed to the disease. The overall survival duration was 9 months. In addition, we drew timelines to better illustrate the current course of the patient’s illness (**Figure 7**).

## Discussion

We present a case of UESL in a 50-year-old woman initially diagnosed with a simple hepatic cyst. The concept and terminology of UESL were first introduced by Stocker and Ishak in 1978 (2). UESL is a highly malignant neoplasm with a poor prognosis, most commonly occurring in children between the ages of 6 and 10 years, although it is rare in adults. Over the past 50 years, fewer than 60 cases have been documented, with patients ranging in age from 25 to 84 years (4, 5). The incidence rate was slightly higher among adult women, and no significant gender disparity was observed in the pediatric population (5).

In general, patients with UESL present with nonspecific clinical manifestations, such as abdominal pain, bloating, fever, diarrhea, or vomiting. Currently, there are no specific serum markers for UESL, and levels of transaminases and tumor markers are typically within normal ranges (6). However, compression of the tumor mass on adjacent hepatic tissue may result in impaired liver function. In cases involving intratumoral hemorrhage or necrotic infection, systemic inflammatory markers—such as white blood cell count, C-reactive protein, and erythrocyte sedimentation rate—may be elevated (7). Imaging findings are variable; ultrasonography commonly reveals a large mass with cystic, solid, or mixed echogenicity (8). CT typically demonstrates well-defined, low-density masses, predominantly cystic in nature, which may contain internal septa and exhibit enhancement following contrast administration (9). MRI typically shows low signal intensity on T1-weighted images and high signal intensity on T2-weighted images. When necrosis or hemorrhage is present, the lesion may display high signal intensity on T1-weighted images and low signal intensity on T2-weighted images (10). Upon reviewing this case, we initially suspected the presence of a malignant tumor. However, the patient’s preliminary laboratory tests did not reveal elevated tumor markers, and both ultrasound and contrast-enhanced CT scans failed to show definitive evidence of malignancy. The only imaging finding suggestive of pathology was suspected intracystic hemorrhage. Intraoperatively, no grossly malignant lesions were observed, and the postoperative pathological examination confirmed a simple hepatic cyst. Despite these findings, the patient was ultimately misdiagnosed with a benign condition, which delayed appropriate management and allowed for subsequent tumor progression. Therefore, greater attention should be directed toward cystic lesions in adults. The following table outlines the pertinent benign and malignant cystic lesions (3, 11–22) (**Table 1**).

**Table 1:** Differential diagnosis of UESL from other cystic disorders in adults.

Type of tumor	Clinical manifestations	Imaging phenotypes	Pathology	Immunohistochemistry
Undifferentiated embryonal sarcoma of the liver	Abdominal mass, abdominal pain, fever and other systemic symptoms	Large cystic or solid mass with internal septa or hemorrhage and necrosis (CT), hypointense (T1-weighted MRI), hyperintense (T2- weighted MRI)	Spindle and stellate pleomorphic cells in a myxoid stroma, brisk mitotic activity, atypical mitoses, eosinophilic globules	Vimentin, α1-antitrypsin, CD68, CD56, Glypican-3
Hepatic mesenchymal hamartoma	Abdominal distention, abdominal mass	Polycystic or mixed mass with well-defined margin, no enhancement (CT), hypointense (T1-weighted MRI), hyperintense (T2- weighted MRI)	Mesenchymal tissue (myxoid matrix), bile duct and hepatocyte components, no malignant characteristics	Vimentin, desmin, SMA
Hepatocellular carcinoma	Abdominal pain, weight loss, AFP rise, abnormal liver function	Arterial phase enhancement, portal venous phase non-peripheral washout (CT), hypointensity (T1-weighted MRI), hyperintensity (T2-weighted MRI)	Hepatocyte differentiation, trabecular and cord-like arrangement, pleomorphic cells, cholestasis	HepPar-1, AFP, CK8/18, CD34
Angiomyolipoma	Asymptomatic or abdominal pain, abdominal mass	Fat-dense mass (low density), mixed vascular and smooth muscle components, heterogeneous enhancement (CT)	Vascular, smooth muscle and adipose tissue are mixed, with no malignant features	HMB-45, Melan-A, SMA, desmin
Gastrointestinal stromal tumor	Abdominal pain, gastrointestinal bleeding, abdominal mass, obstruction	Hypervascular mass, Necrosis or ulcer, Heterogeneous enhancement (CT), hypointensity (T1-weighted MRI), hyperintensity (T2-weighted MRI)	Spindle cells with eosinophilic fibrillary cytoplasm	CD117, DOG-1, CD34
Simple hepatic cyst	Usually asymptomatic; Large cysts may cause abdominal pain, bloating, nausea, and vomiting. Bleeding or infection is rare	Low density, no enhancement (CT), T2 hyperintensity, T1 hypointensity, no enhancement (MRI)	Lined by a single layer of cuboidal or columnar epithelium; The cystic fluid was clear without cellular components. Absence of malignant features	No specificity
Biliary cystadenoma/cystadenocarcinoma	Abdominal pain, abdominal mass, jaundice; Cystadenocarcinoma may be accompanied by systemic symptoms such as weight loss and fatigue	Multilocular cysts with septation and mural nodules; Enhanced scan of cyst wall or septum enhancement (CT); T2 hyperintensity, T1 variable signal, visible enhancement (MRI)	Cystadenoma: simple cuboidal or columnar epithelium with ovarioid stroma (female); Cystadenocarcinoma: epithelial atypia, multilayered, increased mitotic activity, and invasive growth	The epithelial cells were positive for CK7, CK19 and CEA. The tumor stroma may express ER and PR. The Ki-67 index of cystadenocarcinoma was increased
Hepatic hydatid cyst	No symptoms or abdominal pain, hepatomegaly; Rupture of the cyst may cause anaphylaxis (e.g., urticaria, shock). Fever at the time of secondary infection	“Double-wall sign” and “cyst in cyst” structure (ultrasonography); Round low density, cyst wall calcification (CT); T2 hyperintensity, T1 hypointensity (MRI)	Outer capsule (host fibrous capsule), middle layer (cuticle), inner layer (germinal layer); The capsule contained the scolex and asci	It is not usually used for diagnosis; Serologic tests, such as ELISA, are more commonly used

Based on this case and previous case reports, malignant tumors should be considered when the following features are present: first, a large tumor with well-defined margins, containing cystic changes exhibiting heterogeneous density; second, the cystic component shows low density with slightly hyperdense flocculent or cord-like septations or the presence of mural nodules; third, absence of enhancement in the cystic portions, along with inhomogeneous enhancement of the solid parenchyma and septa on contrast-enhanced imaging.

Microscopically, UESL is defined by the presence of spindle-shaped tumor cells and multinucleated giant cells that are diffusely distributed within a myxoid stroma. The lesion also exhibits significant cellular atypia and an increased number of mitotic figures (23). According to the current research findings, Periodic acid-schiff (PAS) staining is recommended for cases of UESL. The detection of PAS-positive eosinophilic inclusions within tumor cells and the surrounding stroma may serve as a potentially specific pathological feature of UESL (24). In terms of immunohistochemical analysis, there is currently no specific immunophenotype associated with UESL. In most cases of UESL, tumor cells demonstrate positivity for vimentin and α1-antitrypsin, along with variable expression of cytokeratin, desmin, α-smooth muscle actin (SMA), muscle-specific actin, CD68, myoglobin, nonspecific enolase, S100, and CD34 (25, 26). The expression of Glypican 3, a fetal oncogenic protein typically present during embryonic development, has been observed in UESL, indicating that UESL exhibits an immature phenotypic profile (27). The immunohistochemical findings from the UESL cases in our study demonstrated positive expression of Desmin, S-100, CD99, and CD68, which are consistent with those reported in previous studies on UESL (28, 29). Furthermore, negative vimentin expression in UESL has been documented in a limited number of cases (30), which is also consistent with our results. We consider this to represent an uncommon immunophenotype; however, it does not entirely exclude the possibility of a diagnosis of UESL. We emphasize that the diagnosis of UESL should be based on a comprehensive evaluation encompassing morphological characteristics, a panel of immunohistochemical markers rather than reliance on a single marker, and the exclusion of alternative diagnoses through integrated assessment. The observed S-100 positivity and vimentin negativity in this case expand the known immunophenotypic spectrum of UESL, underscoring the potential for unexpected immunohistochemical heterogeneity within this tumor entity. In addition, the pathogenesis of UESL remains poorly understood, although it is thought to be associated with specific genetic mutations. Some researchers suggest that UESL may be linked to mesenchymal hamartoma and could represent a malignant transformation of this benign lesion. In this context, genetic alterations involving chromosome 19 are believed to play a critical role, particularly abnormalities at the 19q13.4 locus. Such abnormalities include balanced translocations such as t(11;19)(q13;q13.4) and t(15;19)(q15;q13.4) (24, 31). Cytogenetic studies have identified a diverse array of complex chromosomal abnormalities in individuals with UESL, including gains in chromosomes 1q, 5p, 6q, 8p, and 12q; losses in chromosomes 9p, 11p, and 14 (32); loss of heterozygosity in chromosomes 7p, 11p, 17p, and 22q; and allelic imbalance in chromosomes 1p, 8p, and 20q (33). In addition, some researchers have observed that certain UESL cases exhibit mutations or deletions in tumor protein 53 (TP53), resulting in strong positive expression of p53 in tumor cells (33).

The prognosis of UESL has always been poor. According to the current available studies, children with UESL survive longer than adults, with 5-year overall survival rates of 84.4% and 48.2%, respectively, which may be related to the more aggressive form of UESL in adults. Further studies may be needed to reveal differences in UESL between children and adults and to refine the currently available treatment options for UESL in adults (34). Currently, radical surgery and chemotherapy remain the primary treatment modalities for UESL. Chemotherapy was administered as a combination regimen consisting of vincristine, actinomycin D, ifosfamide, and doxorubicin (23). If complete resection of the tumor is not possible, radical resection after neoadjuvant chemotherapy may be required (35). Liver transplantation is considered a viable treatment option for patients with unresectable or recurrent hepatic tumors, as it is associated with improved long-term survival outcomes (36, 37). The absence of PD-L1 expression in UESL cases indicates that immune checkpoint inhibitors targeting this pathway may have limited therapeutic efficacy in these patients (30). In addition, postoperative tumor recurrence represents a significant challenge for patients with UESL. A study involving 25 patients diagnosed with UESL revealed a postoperative tumor recurrence rate of 32%. Among those who underwent complete tumor resection without receiving adjuvant chemotherapy, 42% experienced tumor recurrence within 8 months following surgery. In contrast, when combination chemotherapy was administered postoperatively, the recurrence rate was reduced to 23%, with a mean recurrence-free survival time of 28 months after treatment (5).

Compared with previous studies, our management of this case remained suboptimal. Initially, the diagnosis was incorrect, and the subsequent surgical intervention failed to achieve complete tumor resection. Although systemic chemotherapy and TACE had been previously reported as potential treatment options (3, 38, 39), the patient opted for TACE; however, the therapeutic response was unsatisfactory, leading to tumor progression and ultimately resulting in the patient’s death. Liver transplantation, a potentially curative option, was not feasible due to financial constraints. We discussed neoadjuvant chemotherapy with the patient, but systemic chemotherapy was declined in favor of upfront surgery. This case underscores the importance of improving diagnostic accuracy for UESL to prevent misdiagnosis and enhance patient outcomes. Treatment strategies should include consideration of neoadjuvant chemotherapy or complete surgical resection combined with adjuvant systemic chemotherapy to optimize prognosis.

## Conclusion

Due to its rare and non-specific clinical manifestations, the diagnosis of UESL remains challenging. It is essential to enhance our understanding of cystic changes associated with malignant liver tumors. The imaging characteristics of hepatic cystic lesions should be thoroughly evaluated during the differential diagnosis of malignant hepatic masses. If a CT examination reveals that the primary tumor capsule is intact, the cyst wall is heterogeneous and thickened, the cystic cavity is compartmentalized, papillary projections extend into the cavity, the tumor demonstrates exophytic growth, the tumor parenchyma is prominent, and hemorrhage is present, a malignant tumor should be considered (40). In suspected cases, multidisciplinary consultation is recommended. If necessary, an ultrasound- or CT-guided biopsy may be conducted prior to surgery to determine the pathological features of the tumor. Intraoperative frozen section analysis plays a crucial role in determining the nature of the tumor, and precise specimen collection is vital to minimizing false-negative outcomes. Furthermore, timely comprehensive treatment, including surgical resection, chemotherapy, radiotherapy, and interventional procedures, can significantly improve patient survival rates and prognosis. Lastly, further research on UESL is warranted to enhance diagnostic accuracy and optimize patient outcomes.

## Data Availability

The raw data supporting the conclusions of this article will be made available by the authors, without undue reservation.
